# Factors influencing conveyance of older adults with minor head injury by paramedics to the emergency department: a multiple methods study

**DOI:** 10.1186/s12873-022-00747-w

**Published:** 2022-11-23

**Authors:** Helen Nicholson, Sarah Voss, Sarah Black, Hazel Taylor, David Williams, Jonathan Benger

**Affiliations:** 1 College of Health, Science and Society, University of the West of England, Glenside Campus (1H14), Blackberry Hill, BS16 1DD Bristol, England; 2 South Western Ambulance Service NHS Foundation Trust, Eagle Way, EX2 7HY Exeter, England; 3 Research Design Service – South West, University Hospitals Bristol NHS Foundation Trust, Upper Maudlin Street, BS2 8AE Bristol, England; 4 Emergency Department, Bristol Royal Infirmary, University Hospitals Bristol NHS Foundation Trust, BS2 8HW Bristol, England

**Keywords:** Head Injury, Minor, Injuries, Head, Aged 65+, Paramedic, Emergency Medical Services, Interview, Decision Making

## Abstract

**Background:**

Head injury (HI) in older adults due to low-energy falls result in a substantial number of emergency department (ED) attendances. However, mortality associated with minor HI is very low. Reducing conveyance to hospital is important for older adults and is a priority for the National Health Service (NHS). Therefore, paramedics are required to make accurate decisions regarding conveyance to the ED. This study used routine data and semi-structured interviews to explore the factors that influence paramedic decision-making when considering whether to convey an adult aged 65 years and over with a minor HI to the ED.

**Methods:**

Semi-structured telephone interviews were completed with ten UK paramedics from a single EMS (ambulance) provider organisation. Interviews explored the factors influencing the paramedics’ conveyance decision-making in adults aged 65 years and over with a minor HI. Data were initially analysed inductively to develop a thematic framework. A retrospective analysis of ambulance service data was also completed to determine the scope and scale of the issue in Southwest England. An in-depth audit of 100 conveyed patient records was used to determine the proportion of patients conveyed to the ED who met National Institute for Health and Care Excellence (NICE) and Joint Royal Colleges Ambulance Liaison Committee (JRCALC) guidelines.

**Results:**

In 2019 South Western Ambulance Service NHS Foundation Trust (SWASFT) attended 15,650 emergency calls to patients aged 65 and over with minor HI, with 70.5% conveyed to ED. 81% of conveyed patients met NICE and JRCALC guideline criteria for conveyance, with the remainder conveyed due to wound care or other medical concerns. The framework developed from the interviews comprised four themes: resources; patient factors; consequences; paramedic factors. Important factors included: the patient’s social situation; guidelines; clinical support availability; the history and presentation of the patient; risk.

**Conclusion:**

This study examined paramedic conveyance decisions for older people with minor HI. It identified multiple influencing factors, highlighting the complex nature of these decisions, and may serve as a basis for developing an intervention to safely decrease ED conveyance in this patient group.

**Supplementary Information:**

The online version contains supplementary material available at 10.1186/s12873-022-00747-w.

## Background

The average age of the United Kingdom (UK) population is increasing with 18% aged 65 and above [1]. This is predicted to increase to 21% by 2030. Head injury (HI) results in 1.4 million emergency department (ED) attendances yearly in England and Wales, most occurring in older persons due to low-energy falls from standing [2]. However, the mortality of HI is very low. The majority of fatalities occur in moderate and severe HIs, which make up only 5% of the total. Most HI patients recover without specific or specialist intervention [3].

Reducing conveyance to hospital is important for older adults and is a priority for the NHS [4, 5]. The NHS Long Term Plan, [4] along with the 2018 Lord Carter Review, [6] aims to improve outcomes and patient experience by supporting the ‘right care, right place, right time’ initiative. The review suggests that reducing avoidable hospital conveyance, particularly for older people, supports delivery of care closer to home, reduces unnecessary pressures on Emergency Departments (EDs) and hospital and could release capacity equivalent to £300 million annually.

It is therefore important for paramedics to make accurate decisions regarding conveyance to the ED, yet most evidence to date has focused on in-hospital management of HI or general paramedic conveyance decision-making.

Older adults have distinct mechanisms of injury, patient characteristics and chronic complications of HI when compared to younger individuals, which requires a unique approach to clinical management and research [7]. This study is intended to investigate the conveyance decisions paramedics make when attending minor HI in older adults. It also assesses adherence to current National Institute for Health and Care Excellence (NICE) and Joint Royal Colleges Ambulance Liaison Committee (JRCALC) guidelines, and may inform the development of future interventions to reduce avoidable conveyance.

For the purposes of this study minor head injury was defined as a pre-hospital Glasgow Coma Score (GCS) of 15 out of 15 [8]. Current NICE HI guidance recommends conveyance to hospital for patients with a GCS of less than 15 [2].

This multiple methods study audited routinely collected ambulance service data and used semi-structured interviews to explore the factors influencing paramedics’ conveyance decision-making in adults aged 65 years and over with a minor HI.

## Methods

Approval for this study was obtained from the Health Research Authority (20/HRA/5970) and the University of the West of England Faculty of Health and Applied Sciences Research Ethics Committee (HAS.20.11.047).

### Setting

South Western Ambulance Service NHS Foundation Trust (SWASFT) is a large emergency medical services (EMS) provider organisation, with responsibility for the provision of ambulance services across an area of 10,000 square miles of South West England, serving a total population of over 5.5 million [9]. The Trust employs over 4,000 clinical and operational staff, and around 3,000 volunteers.

### Data audit

A retrospective analysis of ambulance service data analysis was completed to determine the scope and scale of the issue in Southwest England. The audit used routinely collected data from SWASFT to determine the number of patients aged over 65 years with a minor HI attended, and the proportion of these that were conveyed to hospital, during 2019. An in-depth audit of a random sample of 100 of these conveyed patients was undertaken to determine the proportion of patients conveyed to the ED who met the NICE or JRCALC HI guideline criteria for ED referral.

All data were anonymised prior to analysis. Data were examined in MSExcel© using descriptive statistics. For the in-depth audit a descriptive content analysis was used [10] to examine the anonymised free text areas of the patient record in order to determine the presence or absence of NICE/JRCALC HI conveyance criteria.

### Interviews

Semi-structured telephone interviews with ten operational paramedics working in SWASFT were completed to explore the barriers to and facilitators of non-conveyance of adults aged 65 years and over with minor HI. A phenomenological approach was adopted to inductively examine the subjective experiences of paramedics [11].

### Participant recruitment

Convenience sampling was used, with paramedics recruited through routine ambulance service news bulletins, emails and social media. Paramedics with less than one year’s experience were excluded. Potential participants received information about the study, a consent form and privacy notice via email and verbal confirmation of consent was audio-recorded at the start of the interview. A £10 gift voucher was given to each participant to acknowledge their contribution.

### Design

The number of interviews was not predefined, but was estimated that the topic would be well understood with between 8 and 12 participants. The final sample size (n = 10) was considered adequate as all necessary information was captured as evidenced by no new codes or themes being identified and no further development of codes occurring [12]. If all necessary information was not captured, data collection would be resumed and further interviews undertaken. The topic guide (Additional file 1) was developed by the Study Management Group, including two paramedics, two senior academics in Emergency Care, the SWASFT Head of Research, Audit and Improvement, and a member of the National Institute for Health Research (NIHR) Research Design Service, with input from patient and public involvement representatives. The topic guide was designed to explore participants’ experiences of attending older adults with minor head injuries, and decision-making around conveyance to ED. Audio-recordings of the interviews were made using Skype for Business. Recordings were transcribed verbatim and anonymised.

### Qualitative data analysis

At the end of data collection, data were analysed thematically with inductive coding. Transcripts were imported into the data-management software NVivo 10 and read several times. Sections of text were then coded to represent instances of a concept [13]. Codes were reviewed and combined to develop themes. A framework for the themes was developed from the inductive analysis. Interview data were coded by one person, however, a sample of 20% was checked by another member of the study team and any areas of uncertainty were discussed and agreement reached. One researcher selected supporting evidence to represent each theme and provide examples of how the theme influenced the conveyance decisions of paramedics.

## Results

### Data audit

In 2019 SWASFT attended 15,650 emergency calls to patients aged 65 and over with minor HI. This represented 79% of all HI calls to older people and the average age was 83 years. Of these, 70.5% were conveyed to an emergency department and 1.6% were conveyed to other destinations such as a minor injury unit (MIU), community hospital or hospice.

The in-depth audit found that 81% of patients conveyed to the ED met the NICE and JRCALC HI guideline criteria for conveyance. Of the 19% with an absence of documented NICE or JRCALC conveyance criteria, wound care accounted for half and the other half were noted to have underlying medical problems. Further details of electronic patient clinical record (EPCR) completion and patient characteristics can be seen in Table 1.

**Table 1:** Ambulance electronic patient clinical record completion and patient characteristics

	N (*n* = 99^a^)
HI tool indicated conveyance	50
HI tool did not indicated conveyance	8
Incomplete or absent HI tool	41
Clinical Frailty Scale (CFS) completed	78
CFS ≥ 5	32
CFS ≥ 7	7
Female	52

^a^1 patient was excluded as a GCS of less than 15 was mentioned in the free text section of the electronic patient record. Patients aged 65 years and over with a minor head injury conveyed by ambulance in 2019

### Interviews

#### Participant characteristics

Ten operational paramedics took part in semi-structured telephone interviews. The majority of participants were male (70%). Participants represented the full range of paramedic qualification routes, and experience as a qualified paramedic ranged from 1.5 years to 27 years (mean ± SD, 8.55 ± 8.85). Participants roles included: Paramedic (6); Newly Qualified Paramedic (NQP) (2); Specialist Paramedic (1); and Remote Clinical Validation (1).

### Key themes

The framework developed comprised four main themes: resources; patient factors; consequences; paramedic factors (Additional files 2, 3, 4 and 5).

#### Resources

This theme was the most prevalent and included three subthemes: social situations and safety netting; guidelines; clinical support. It was a particularly rich theme with all three subthemes represented across all transcripts. It covered resources accessible to paramedics to support decision-making as well as those available to the patient such as the availability of family or carers.

Social situation and safety netting was the most common subtheme and dealt with issues such as the difficulties encountered by paramedics when attending patients who live alone or who are socially isolated. For these patients, safety was a major factor in the participants’ decision making. This included concerns over whether the patient could care for themselves, mobilise safely, comply with ongoing minor wound care, understand HI worsening care advice and call for further help if needed. Not having someone available to check on the patient and ring back if their condition worsened was often the biggest barrier to non-conveyance. This was linked to one of the possible facilitators suggested by participants which focused on the ability to organise a follow up mechanism for the patient over the coming hours or days to check in on them. Participants also found that not having an available witness to confirm the history of events or whether the patient was behaving normally was a barrier.

Despite the patient’s social situation being such an influential factor in decision-making, only 43% of conveyed patients had information about their social circumstances recorded in their EPCR. Of those with details recorded, 37% lived alone and 23% lived in a residential care or nursing home.

Guidelines was the second most prominent subtheme. This covered areas such as anticoagulants, use of a head injury assessment tool embedded in the electronic patient record and the NICE HI guidelines. In general, participants found the guidelines to be clear, and felt that having a list of ‘red flags’ in the head injury tool was useful. However, participants also found the guidelines to be somewhat restrictive at times, with a tendency to limit paramedic autonomy and a bias towards conveyance. This was sometimes at odds with what the participants thought was in the best interests of the patient. Some participants reported that the NICE HI guidelines were less appropriate for frail patients where participants thought that conservative management of any possible HI would be more likely.

Participants reported trying to balance guideline adherence with a more holistic and patient-centred approach to conveyance decisions. Four of the participants thought the guidelines should support their decisions rather than dictate them, and that individual circumstances should be considered. However, one of these participants also reported that they would rarely deviate from the guidelines and admitted to conveying some patients more because of guidelines than because of perceived risk.

Anticoagulants was a particularly well-represented topic within the guidelines subtheme, and appeared in all transcripts. All participants recognised that the guidelines recommended conveyance for patients taking anticoagulants. However, some reported confusion as to which medications were included, and whether anti-platelet drugs were also a justification for ED conveyance. This was supported by the audit data which showed that around half of the patients who were conveyed due to anticoagulants were actually taking the antiplatelet drug Clopidogrel, but no anticoagulants.

The clinical support subtheme covered topics including being able to discuss with other clinicians, accessibility of services, referrals and wound care. Additional clinical support was a facilitator to non-conveyance when available, and a lack of availability was frequently mentioned as a barrier. Participants reported that patients were often conveyed to ED because the preferred alternative pathway was either closed or unable to accept the referral. Time of day was therefore an important factor in decision-making.

A lack of confirmation or feedback from some referral pathways meant that participants felt unsure if patients would be followed-up and within what timeframe, thereby reducing confidence in the level of clinical safety achieved. Suggested facilitators (Additional file 6) included a follow-up pathway with a guaranteed patient call back from a clinician within 12 h.

Wound care was frequently mentioned as a cause of conveyance, often exacerbated by the inaccessibility of alternative out of hours services. Several participants reported that they did not have the training or equipment needed to undertake wound closure. One participant with extended wound care skills felt that it was ‘a huge barrier [to non-conveyance] out of the way’ when they could deal with small wounds at home.

Being able to talk to a senior clinician was seen as a facilitator of non-conveyance. Participants found this especially helpful when stepping outside of guidelines in order to meet individual patient needs. Electronic referrals were less reassuring as participants were unsure whether it was ever followed up by someone due to a lack of feedback mechanisms.

#### Patient factors

This was another rich theme represented across all transcripts. It had two subthemes: history and presentation; patient or family preferences. History and presentation focused on comorbidities, underlying causes for falls, frailty, mechanism of injury and “red flags”. The presence of any “red flags” (NICE HI conveyance criteria), concerning underlying illness or comorbidities was an important barrier for many of the participants when considering non-conveyance. A concerning or unclear mechanism of injury was also a barrier to non-conveyance.

The patient and family preferences subtheme included issues such as patient refusal/choice, patient distress and advance treatment plans. The patient’s preferences were an important factor, especially when they were less keen to go to hospital or refused transport. A clear advance care plan was seen as facilitating non-conveyance. However, some plans were found to have ‘grey’ areas which one participant felt could lead to increased conveyance, for example, when a patient’s advanced care plan states that the patient is ‘not for hospital’ but the exceptions listed are not well defined.

#### Consequences

This theme included two subthemes: risk; repercussions and hospital/trust support. The subtheme of risk encompassed risk aversion, the patient’s best interests, the risk/benefit balance, patient age and “scare stories”. Many participants described risk aversion in relation to non-conveyance with a “better safe than sorry” or “not worth the risk” approach leading to some conveyances. This risk aversion was reported to be exacerbated by “scare stories” about patients with poor outcomes during training or clinical updates.

The balance of risk and benefit was an important factor, and this related to risk for both the paramedic and the patient. For patients the benefit of non-conveyance was often thought of as reduced patient distress, especially for dementia patients. Participants felt that many patients were able to weigh up the risks and benefits and may choose to accept the risks of remaining at home. However, for participants the balance was heavily weighted towards the risk of a patient having a poor outcome after being left at home and the paramedic being held responsible. This risk/benefit balance was reported to be very patient specific.

The other subtheme of repercussions and trust/hospital support was closely related to the subtheme of risk and was comprised mostly of barriers to non-conveyance. It included factors such as hospital opinion, the paramedic governing body (Health and Care Professions Council (HCPC)), uncertainty and clinician validation. Repercussions were spoken about in terms of formal discipline from the trust or governing body (HCPC) as well as judgement from other clinical staff, particularly staff at receiving hospitals or from senior clinicians. Participants also feared the anticipated stress of an investigation should a patient have a poor outcome following non-conveyance.

#### Paramedic factors

This theme included two subthemes: confidence; experience. More experience was associated with greater confidence to non-convey. Confidence was also linked to self-reflection and further education or training. A lack of feedback as to whether previous conveyance decisions were correct reduced confidence, though it was generally assumed that an adverse outcome would quickly be fed back to the paramedic.

#### Suggested facilitators

Potential facilitators suggested by participants are shown in Additional file 6. These included: enhanced training; wound closure skills and equipment; less emphasis on worst case scenarios; more room for clinical judgement in guidelines; a follow up referral pathway.

## Discussion

This research addresses one of the top fifteen NIHR research priorities for emergency medicine, ‘the prediction of which older patients attended by an ambulance crew can be safely and effectively managed at home’ [14]. Previous observational studies have explored the characteristics of EMS transport decisions for older patients that have fallen, [15–17] and one qualitative study described the complexity of these decisions [18]. However, this paper is believed to be the first to explicitly explore paramedic conveyance decision-making for minor HIs in older adults, meaning that results are discussed here in the context of a wider patient population. The most frequently mentioned influencing factors in this study were centred around resources, the history and presentation of the patient and risk.

Previous research on paramedic conveyance decisions for persons with dementia found that the person’s clinical condition was the key factor [19]. The patient’s circumstances, including the support available to them at home, were also recognised as important which reflects the results of this study. In particular, living alone was an important barrier to non-conveyance, and this applied to 37% of the patients for whom social details were recorded.

All areas described in the history and patient presentation subtheme (comorbidities, frailty, “red flags”) are factors known to increase the risk of a poor patient outcome for older people with HI [7] or for older people in general [2, 20, 21]. It is therefore unsurprising that they were so well represented and that their presence resulted in participants being less likely to non-convey. A systematic review of general conveyance from care homes by Marincowitz et al. also found that comorbidities such as dementia or increased frailty were associated with increased transfer to the ED [22], although none of the included studies were conducted in the UK. However, these pertinent clinical factors balanced against risk aversion and the fear of repercussion described in the current study may result in an overly cautious approach to non-conveyance. The weighing-up of risks and benefit for both the clinician and the patient that was described by participants is similar to that undertaken by staff in care homes when deciding on hospital transfer for older people generally [23].

Participants sometimes found that balancing guidelines and the best interests of the patient could be challenging. This mismatch of guidelines and clinical opinion has been found in previous research on UK paramedic conveyance decisions [19, 24]. In particular, some participants reported they felt the NICE HI guidelines were less appropriate for frail patients. Frailty is prognostic within the general older adult population, with frail patients (those with clinical frailty score (CFS) of 5 or more [25, 26]) having higher in-hospital and long-term mortality [20] and being less likely to be discharged home than non-frail patients [21]. The NHS Specialised Clinical Frailty Network suggests that care intentions should consider palliative rather than restorative care for patients with a CFS of 7 or more [27], which would apply to 9% of the conveyed patients with a CFS recorded in this audit.

Being able to discuss with other clinicians was a facilitator of non-conveyance. The option of additional clinical support appeared very popular in one United States (US) study where paramedics consulted a physician in 82% of falls even when the protocol deemed transport and consultation unnecessary [28].

Risk played an important role in participants decision making both in terms of risk to the patient but also the perceived risk of a poor patient outcome and the paramedic being held responsible resulting in legal, reputational and/or career repercussions. For these reasons participants felt that it is important to be able to defend conveyance decisions and in general, they felt it is safer and sometimes easier to take a patient to hospital than risk leaving them at home or trying to arrange follow up. However, a cluster randomised controlled trial in the UK found that paramedics with extended skills were able to treat older people with minor injury or illness at home just as safely as transfer and treatment within an ED [29]. Amongst the participants in the current study there was an appetite for extended training and in particular wound care skills. There is also evidence to suggest that older people without additional risk factors (such as antiplatelet therapy) under the age of 80 could be managed as younger patients with only 0.66% of 65-79-year olds with a minor HI having pathological findings on their CT scan [30]. Further evidence suggests that even anticoagulated patients are at low risk of an adverse outcome if they have no symptoms and a GCS of 15 [31].

This study suggests that any future interventions hoping to reduce ED conveyances would need to consider the availability of social support and alternative pathways, but also mitigate risk aversion by providing paramedics with a robust ethical and legal framework to support these complex decisions.

## Study limitatations, strengths and future directions

The number of participants was small but represented a good range of paramedic experience and education routes. Despite the small number of participants, all necessary information was captured as evidenced by no new codes or themes being identified and no further development of codes occurring [12].

All participants were from a single EMS provider organisation which may have a different culture and practices compared to other EMS providers in the UK and internationally.

In this study minor HI was defined as a GCS of 15. However, this excluded head injury patients with a reduced GCS due to dementia or other causes. This was due to the difficulty of distinguishing these patients from those with a reduced GCS due to a more severe HI.

No safety outcome data were collected. This will be an important area for further research in order to assess the appropriateness of conveyance decisions.

## Conclusion

This study demonstrates the complexity of paramedic conveyance decision-making for older adults with minor head injury. Key factors are similar to those that influence paramedic conveyance decisions generally, and include the availability of social support or alternative services for the patient, the balance between guidelines and paramedic autonomy, the history and presentation of the patient and the risk/benefit balance. However, for older people with minor HI, the patient’s social situation plays the dominant role in paramedic conveyance decision-making, often due to the period of observation that was felt to be needed to reliably identify deterioration in this patient group.

The influencing factors identified may serve as a basis for developing an intervention to safely increase non-conveyance in this patient group. This research has the potential to inform clinical practice in the short term and could have a longer-term impact on reducing hospital admissions.

## Supplementary Information

**Additional file 1.** Interview topic guide. Topicguide for paramedic interviews.

**Additional file 2.** Resource factors influencing conveyance of olderadults with minor head injury by paramedics.

**Additional file 3.** Patientfactors influencing conveyance of older adults with minor head injury byparamedics.

**Additional file 4.** Consequence factors influencing conveyance ofolder adults with minor head injury by paramedics.

**Additional file 5.** Paramedic factors influencing conveyance ofolder adults with minor head injury by paramedics.

**Additional file 6.** Suggested facilitators of non-conveyance ofolder people with minor head injury by paramedics. 

## Data Availability

The datasets generated and analysed during the interview study are not publicly available due to participant confidentiality, but are available from the corresponding author on reasonable request.

## Abbreviations

CFS: Clinical frailty scale
ED: Emergency department
EMS: Emergency medical service
EPCR: Electronic patient clinical record
GCS: Glasgow Coma Score
HCPC: Health and Care Professions Council
HI: Head injury
JRCALC: Joint Royal Colleges Ambulance Liaison Committee
MIU: Minor injury unit
NHS: National Health Service
NICE: National Institute for Health and Care Excellence
NIHR: National Institute for Health Research
NQP: Newly Qualified Paramedic
SWASFT: South Western Ambulance Service NHS Foundation Trust
UK: United Kingdom
