# Supplementary material for: Factors influencing conveyance of older adults with minor head injury by paramedics to the emergency department: a multiple methods study
Source: BMC Emerg Med. 2022 Nov 23;22:184. doi: 10.1186/s12873-022-00747-w (PMC9682699; doi:10.1186/s12873-022-00747-w)
Supplement: Supplementary file 1 — Additional file 1. Interview topic guide. Topicguide for paramedic interviews. [file 12873_2022_747_MOESM1_ESM.pdf]

Minor Head Injury Interview Guide

The interview guide will be agreed by the study management team and PPI representatives before data collection.

Introduction: Thank you for agreeing to take part in our study. We are conducting interviews with paramedics to understand your experiences of attending adults aged 65 years and over with minor head injuries and the factors affecting your conveyance decisions. I'll be asking you open-ended questions. There are no right answers. You are the expert about your thoughts and experiences, and I'm here to learn from what you have to say. This is a chance for you to talk in depth, and I encourage you to tell me as much as you can and use examples, because that is the kind of data that is the most useful for us in answering our research question.

The interview will last approximately 30 minutes. All that you say will be recorded using an audio-recorder. However, this is so that it can be typed up. The recording will not be played to anyone else, except the typist. It will be destroyed after the study is complete. Your interview will be given a PIN (participant identification number) so you cannot be identified by anyone else. You are being given a gift voucher to the value of £10 to acknowledge the time you have given to participate. This does not mean you have to answer all the questions, and you can change your mind and withdraw from the research at any time if you wish.

1. Opening

   a. Could I start by asking how long you have been a paramedic and how you were trained?

2. Identifying patients who can be safely non-conveyed.

   a. What would you consider to be a minor head injury?

For the purposes of this study we are using the definition of a GCS of 15 to indicate a minor head injury.

   b. Can you remember an experience in which you were considering whether or not to non-convey an older adult with this kind of head injury? (ask for detailed description)

   c. What factors influenced your decision making when deciding if they could be non-conveyed?

   d. How easy do you think it is to identify an older adult who can be safely non-conveyed?

   e. Do you feel there are any barriers to non-conveyance for these patients?

   f. Is there anything that you feel would help you to identify patients who can be safely non-conveyed?

   g. What pathways, if any, are available/have you used when non-conveying these patients?

3. Head injury guidelines

a. Are there any guidelines or tools that you use when making conveyance decisions for these patients?

4. Summary

   a. It sounds as if…… would this be a correct summary of the things you have told me?

   b. Do you have any particular thoughts about clinical guidance in relation to head injury research that you would like to discuss now?

   c. Is there anything that you think is relevant that we haven't covered?

Thank you for your participation.
