# Supplementary material for: Factors influencing conveyance of older adults with minor head injury by paramedics to the emergency department: a multiple methods study
Source: BMC Emerg Med. 2022 Nov 23;22:184. doi: 10.1186/s12873-022-00747-w (PMC9682699; doi:10.1186/s12873-022-00747-w)
Supplement: Supplementary file 2 — Additional file 2. Resource factors influencing conveyance of olderadults with minor head injury by paramedics. [file 12873_2022_747_MOESM2_ESM.docx]

**Additional file 2 - Resource factors influencing conveyance of older adults with minor head injury by paramedics**

| **Theme** | **Subthemes** | **Supporting evidence** |
| --- | --- | --- |
| **Resources** | **Social situation and safety netting**  (Living alone, patient support available, follow up) | *In the absence of anybody that knows them well it can be difficult because we’re not going to know if the way they present is normal for them or not. (P001)*  *[If] they are somebody who I am socially quite concerned about……. I’d probably have a lower threshold of taking those people to secondary care…. (P002)*  *When we say goodbye to a patient, we very rarely hear anything about them again, unless they call again. So it's very difficult for us to be sure that everything we're putting in place actually happens. Because we'll never find out, and so there's always that doubt in your head…. (P004)*  *…are they safe alone, or safe in the home environment, is definitely a big factor as well. (P005)*  *If nobody is there to monitor them then that’s always going to be a risk, so I would see that in it, that as a barrier [to non-conveyance]. (P006)*  *…for me when it’s an older person always kind of comes down to safety netting. (P009)*  *I think you know we were concerned about how he would present back to healthcare services if he were to develop any red flag symptoms. So from that point of view we had to convey him…. (P009)* |
|  | **Guidelines**  (Anticoagulants, head injury tool, NICE guidelines) | *…so the hospital therefore give you the impression that they don’t really want them in, and we’re conveying them because of a guideline rather than it necessarily being in the patient’s best interests…. (P001)*  *…[head injury and c-spine tools] are obviously very useful and there for a good reason but they are quite, I suppose they are quite heavy on recommending conveyance…. (P002)*  *….[the head injury tool is] very rigid and obviously everything's grey really, in reality. (P004)*  *I think the guidelines are pretty good generally…… but they will push you to admitting a patient, rather than not. (P004)*  *…..I think the important thing is to remember that they are guidelines and they’re there to support rather than to dictate. (P008)*  *I think the guidelines aren’t very patient specific at the moment, I think they just kind of you know give everyone this blanket, you know yes, no…. (P009)*  *I think there’s a subset of patients where there’s you know could be consideration towards more conservative management and I don’t think the NICE guidance really hit those patients very well. (P009)*  ***Anticoagulant specific:***  *I’m sure over our careers we could have taken 85-year-olds in on anti-coagulants and left a risky 56-year-old who actually is the one that goes on and has the bleed. (P001)*  *…so a big thing in the older population is whether they are anticoagulated… (P002)*  *….the most recent examples would be patients that are otherwise well and the only reason for taking them is because of the medication that they are taking… (P003)*  *One thing that I’ve found confusing in the guidelines around here as well is Clopidogrel… they say consider Clopidogrel and Apixaban and I know all the NOACs definitely get scanned but Clopidogrel seems to be a grey area… (P007)*  *We were considering not conveying her but on review of her medicines she was on clopidogrel so we went on the side of caution to convey her instead just because of the anti-platelet therapy that she was on. (P010)* |
|  | **Clinical support**  (Able to discuss, referrals, accessibility, wound care) | *So a lot of it can depend on kind of time of day, time of weekend and place where you are. (P002)*  *I find it easier to sometimes discuss it with somebody in the whole context of things rather than some things just tick boxing yes or no. (P002)*  *So I think sometimes yes seeking medical support can be a good way round some of that kind of uncertainty or worry perhaps about going against the guidance for some patients. (P003)*  *So sometimes quite often in the out of hours setting, A&E is the only alternative you have, especially if it's a minor injury.. (P004)*  *However, if you're outside of those borders, that service isn't available and it can take days to get a physiotherapist from a community team, out to see a patient. (P005)*  *…there have been occasions previously where it’s, I’ve taken patients to hospital, especially with head injuries that it is purely for wound care or wound closure. (P006)*  *…when it’s at night time, you can send off all these referrals either to the GP or anyone else but you don’t really have reassurance that that would definitely reach somebody. (P007)*  *I’ve actually done my SWAMP [wound care] training, so at least now I can close small head wounds at scene……so I feel that’s a huge barrier out the way, when you can deal with a small wound at home. (P007)*  *….a local hospital has just brought out a specific elderly person advice line…….. which is really, really great, really good service. (P005)* |
