# Supplementary material for: Factors influencing conveyance of older adults with minor head injury by paramedics to the emergency department: a multiple methods study
Source: BMC Emerg Med. 2022 Nov 23;22:184. doi: 10.1186/s12873-022-00747-w (PMC9682699; doi:10.1186/s12873-022-00747-w)
Supplement: Supplementary file 3 — Additional file 3. Patientfactors influencing conveyance of older adults with minor head injury byparamedics. [file 12873_2022_747_MOESM3_ESM.docx]

**Additional file 3 - Patient factors influencing conveyance of older adults with minor head injury by paramedics**

| **Theme** | **Subthemes** | **Supporting evidence** |
| --- | --- | --- |
| **Patient factors** | **History and presentation**  (Comorbidities, underlying causes, frailty, mechanism of injury, red flags) | *I think for me it’s about breaking the story down, trying to work out for myself what’s happened, and visualise that in my mind. (P001)*  *I think the other factor that often crops up is perhaps their general state, their frailty and their sort of general quality of life. (P003)*  *I've got to be happy that there is not something else underlying that's caused them to fall in the first place. (P004)*  *Obviously if they had no red flags and were well in themselves and they didn’t have any concerns or factors I would be more confident in discharging them… (P010)* |
|  | **Patient or family preferences**  (refusal, choice, advance care plans) | *….for every patient that’s keen to go to hospital, a lot of them don’t want to because it’s a hassle getting back home again, so they’re looking to us to make a safe decision to hopefully prevent them going, I think a lot of them are. (P001)*  *….some of these non-conveyances might not be because I don't want them to be, because I want them to go to hospital, but they're refusing….. (P004)*  *….a lot of people have advanced care plans…… and it's so grey, it sort of pushes everything back into taking them to hospital again. (P004)*  *….each of us has to always be incredibly aware of is the patient’s choices as well, their right to choose whether to go or not and, you know, if a patient is absolutely insistent that they need to be seen in an A&E department, sometimes we’re going to take them in…. (P008)* |
