# Supplementary material for: Factors influencing conveyance of older adults with minor head injury by paramedics to the emergency department: a multiple methods study
Source: BMC Emerg Med. 2022 Nov 23;22:184. doi: 10.1186/s12873-022-00747-w (PMC9682699; doi:10.1186/s12873-022-00747-w)
Supplement: Supplementary file 4 — Additional file 4. Consequence factors influencing conveyance ofolder adults with minor head injury by paramedics. [file 12873_2022_747_MOESM4_ESM.docx]

**Additional file 4 - Consequence factors influencing conveyance of older adults with minor head injury by paramedics**

| **Theme** | **Subthemes** | **Supporting evidence** |
| --- | --- | --- |
| **Consequences** | **Risk**  (age, best interests, risk aversion, risk benefit balance, scare stories) | *There will always be a few that will need to go in, just because it’s better safe than sorry… (P001)*  *Some people just take all of them in, regardless of whether they're over 65, but generally it's over eighties that might get the blanket conveyance. (P004)*  *I find that sometimes it feels easier to take them to hospital where a CT scan could be done and the patient is obviously observed for a few hours which obviously then reduces the risk for that patient and to sort of yourself being questioned later on. (P010)*  *I think we are quite exposed pre-hospitally, you know, we don’t have the options of to sit in watching and waiting and kind of observing for a couple of hours. (P002)*  *I think a lot of the time we just think well it is not really worth the risk, even though there’s no visible head injury, it doesn’t sound like you’ve really banged your head very hard because the guidance suggests that you should and there’s no real clear reason not to send him in… (P003)*  *….where our training comes in, we have updates, we start getting these sort of scare stories come in that you need to be aware of, but they heavily influence you, against leaving people at home. (P005)*  *…..it's really difficult, it's your career, it's your livelihood, and of course it's someone's life as well, so you have to be really careful with it. (P004)*  *….there’s always an element of scaremongering around head injuries and being really cautious about them. (P009)* |
|  | **Repercussions and Trust/hospital support**  (HCPC, hospital opinions, uncertainty, NQP validation) | *…..NICE tell us to take patients in but then the hospitals don’t always do something with them either, so the hospital therefore give you the impression that they don’t really want them in…. (P001)*  *….there’s always that fear of investigation and punitive action that may follow the day that you get one wrong, which, inevitably I guess we all might do one day. (P001)*  *I definitely found that sometimes is a bit of a barrier to discharging because you sort of feel that you are taking on a lot of risk and that you are almost being hung up to dry I guess. (P010)*  *I think that we all have that in the back of our mind, how am I going to defend myself if I end up in front of the HCPC, with this? (P004)*  *….if I go to the patient, they have a head injury, I leave them at home, and they're over 65 years old, and they end up dying, that will come directly back to me. I'll be suspended from duty, they'll notify the HCP, and it could take months and months to go through that stress, from someone that it would have taken 20 minutes to get to hospital………. it is a major problem and major fear factor for me, leaving the older people, elderly people at home with a head injury. (P004)*  *….most people say you'll never get the sack for taking someone to hospital. And … and a lot of people live by that…. (P004)*  *You want to get to your … the end of your career, without any … without ever having to be in front of your governing body. (P004)*  *….the more co-morbidities they have, the more nervous you get of leaving them at home, because they're more likely to die, it might be totally unrelated to what you've seen them for, but it might just be their time, but again, if they die the next day, even if it's unrelated, you know you're going to be in the dock for it. (P004)*  *…you’re trying to discharge a head injury you are trying to think about what’s safe for the patient but you are also trying to think about is the validator going to go for this, you know? (P009)* |
