# Supplementary material for: Factors influencing conveyance of older adults with minor head injury by paramedics to the emergency department: a multiple methods study
Source: BMC Emerg Med. 2022 Nov 23;22:184. doi: 10.1186/s12873-022-00747-w (PMC9682699; doi:10.1186/s12873-022-00747-w)
Supplement: Supplementary file 5 — Additional file 5. Paramedic factors influencing conveyance ofolder adults with minor head injury by paramedics. [file 12873_2022_747_MOESM5_ESM.docx]

**Additional file 5 - Paramedic factors influencing conveyance of older adults with minor head injury by paramedics**

| **Theme** | **Subthemes** | **Supporting evidence** |
| --- | --- | --- |
| **Paramedic factors** | **Confidence** | *…the more experience you’ve got, the more confidence you’ve got to say you’re probably going to be fine to stay here today. (P001)*  *…where I get an advanced practice level and having done Masters level education in patient assessment and diagnostics and that kind of thing, but that definitely helps with my ability and confidence with decision making…. (P003)*  *….there’s not always that feedback loop to know that I have left someone safely there. (P007)*  *I know I would hear if anything drastic happened, but I would assume that most of the people I’ve identified and remained at home have been okay, have been identified correctly. (P007)* |
|  | **Experience** | *I think with the experience you get better at questioning in more detail, and just trying to get to the bottom of what actually might have happened really. (P001)*  *Perhaps I operate slightly differently to other paramedics with perhaps less experienced paramedics and that I rely on my experience and additional training. (P003)*  *But I do notice in a lot of the younger paramedics, especially the newer ones, they … they leave a lot of people at home that I wouldn’t… (P004)*  *The basic assessments, if it’s good, it should lend it one way or another really and, experience of people, and your own experience is always playing to that. (P008)* |
