# Supplementary material for: Factors influencing conveyance of older adults with minor head injury by paramedics to the emergency department: a multiple methods study
Source: BMC Emerg Med. 2022 Nov 23;22:184. doi: 10.1186/s12873-022-00747-w (PMC9682699; doi:10.1186/s12873-022-00747-w)
Supplement: Supplementary file 6 — Additional file 6. Suggested facilitators of non-conveyance ofolder people with minor head injury by paramedics. [file 12873_2022_747_MOESM6_ESM.docx]

**Additional file 6 - Suggested facilitators of non-conveyance of older people with minor head injury by paramedics**

| **Theme** | **Subthemes** | **Supporting evidence** |
| --- | --- | --- |
| Training | Enhanced training | *…an increase in sort of training and knowledge I guess would be of benefit if there was to be a training package that maybe would sort of make me more comfortable in identifying patients that might be safer to discharge. (P010)*  *…probably more urgent care work and urgent care kind of focus in the ambulance service…… I think the difference in what we see in the ambulance service as normal head injuries and what we see in urgent care or primary care as normal head injuries, there is a completely different emphasis on how to treat. (P002)*  *I think in terms of the wider paramedic population, having those [SP] skills and knowledge and ability would be an enabling factor and increasing the number of patients that aren’t conveyed to hospital. (P003)* |
|  | Wound closure skills and equipment | *…think if there was more emphasis on wound closure, you know, they have blast dressings, they have all this stuff for big wounds, but you very rarely see those. But we have virtually nothing for small wounds. (P004)*  *…training in minor injury closure, and pathways would … would really help us I think. (P004)*  *…the SWAMP courses that SWAFT run are starting to get back up and running and I think that will, with certain aspects of head injuries hopefully will reduce the amount of conveyances. (P006)* |
|  | Less emphasis on worst-case scenarios | *…perhaps in training it needs to be a little bit more focused on the realities of what we are most likely to see rather than the worst-case scenarios of what we sometimes see. (P002)*  *And recent training, we've had a bit of a scare, with aspirin and Clopidogrel, so they say well, it's up to you if you leave somebody at home. But this is a case study of where somebody was left at home, they took, aspirin, two weeks later, they called an ambulance back and they've had a slow bleed. They've got a GCS of five, and I think they died. (P004)* |
| Guidelines | Clarity and more room for clinical judgement | *It’s just having a little bit of wriggle room, it’s probably not the right phrase to use but if you have a lot of yes, no, or you must do this and you must do that, it’s very difficult to exercise your judgement, isn’t it? (P001)*  *...they’ve hit their head and they’re on anti-coagulant they have to go in, so for me just changing the wording of that might help a little bit. (P001)*  *I think that some of the guidance….. is perhaps focused very strongly on worst case scenario and yes, whether that is helpful for the vast majority of patients or not, I don’t know. (P002)*  *I think there’s a subset of patients where there’s you know could be consideration towards more conservative management and I don’t think the NICE guidance really hit those patients very well…..That’s kind of the group of patients that probably be dealt with better pre-hospitally. (P009)* |
| Follow up | Follow up with a clinician within a given timeframe | *…maybe put people into the minor injuries unit, the following day, or three days down the way, whatever it was, that would be helpful I think that's probably the biggest thing. It's always safety netting, and then you would be happier with your decision making then, providing you've got some ongoing care. (P004)*  *I think what could be good is a sort of clinician call back the next day from a paramedic or somebody at a clinical desk just to follow up and check they’re okay. I think, if you knew perhaps sometimes that someone is going to definitely check in with the patient in 12 hours to say, just to check that everything’s okay that would give you a bit more reassurance, perhaps if you’re feeling that it’s a little borderline. (P007)* |
